# Supplementary material for: Enhancing Efficiency in Inverted Quantum Dot Light-Emitting Diodes through Arginine-Modified ZnO Nanoparticle Electron Injection Layer
Source: Nanomaterials (Basel). 2024 Jan 26;14(3):266. doi: 10.3390/nano14030266 (PMC10856329; doi:10.3390/nano14030266)
Supplement: Supplementary file 1 [file nanomaterials-14-00266-s001.zip › nanomaterials-2820091-supplementary.pdf]

Supporting Information

Enhancing Efficiency in Inverted Quantum Dot Light-Emitting Diodes through Arginine Modified ZnO Nanoparticle Electron Injection Layer

Young-Bin Chae, Su-Young Kim, Hyuk-Doo Choi, Dae-Gyu Moon, Kyoung-Ho Lee*, and Chang-Kyo Kim*

[1]Department of Electronic Materials, Devices, and Equipment, Soonchunhyang University, Asan, Chungnam 31538, Republic of Korea

*Corresponding Author Email: khlee@sch.ac.kr (Kyoung-Ho Lee); ckkim1@sch.ac.kr (Chang-Kyo Kim)

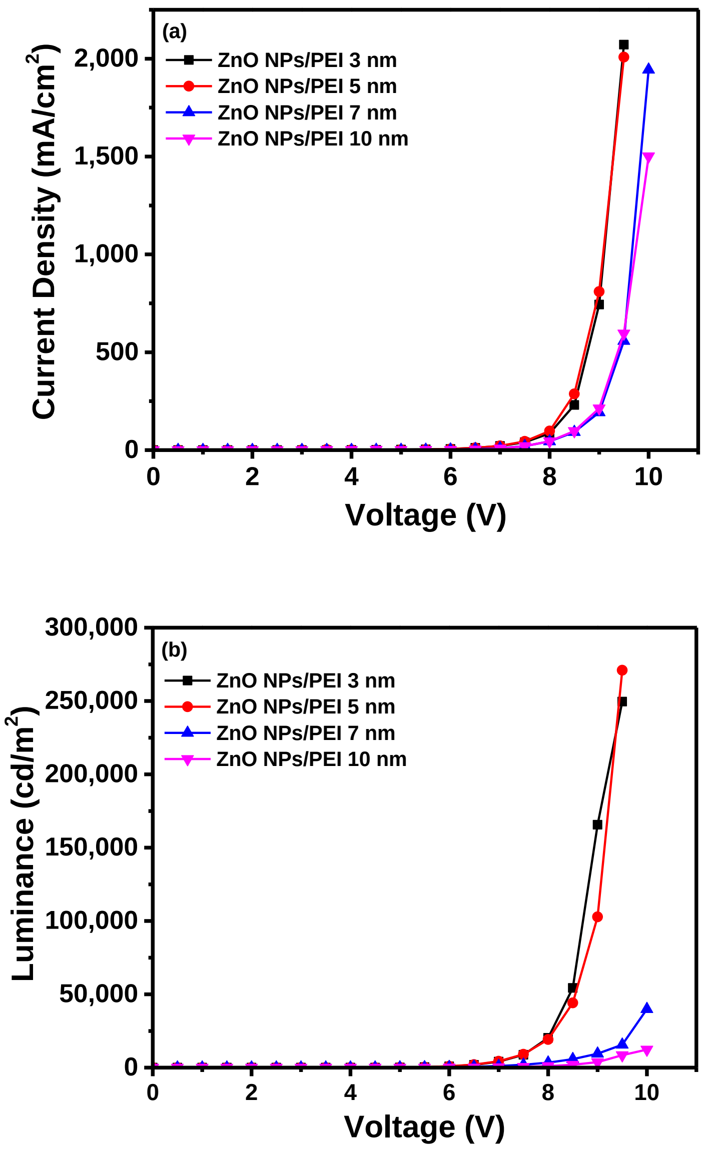

(a)
ZnO NPs/PEI 3 nm
ZnO NPs/PEI 5 nm
ZnO NPs/PEI 7 nm
ZnO NPs/PEI 10 nm
Current Density (mA/cm2)
2,000
1,500
1,000
500
0
0
2
4
6
8
10
Voltage (V)
(b)
ZnO NPs/PEI 3 nm
ZnO NPs/PEI 5 nm
ZnO NPs/PEI 7 nm
ZnO NPs/PEI 10 nm
Luminance (cd/m2)
300,000
250,000
200,000
150,000
100,000
50,000
0
0
2
4
6
8
10
Voltage (V)

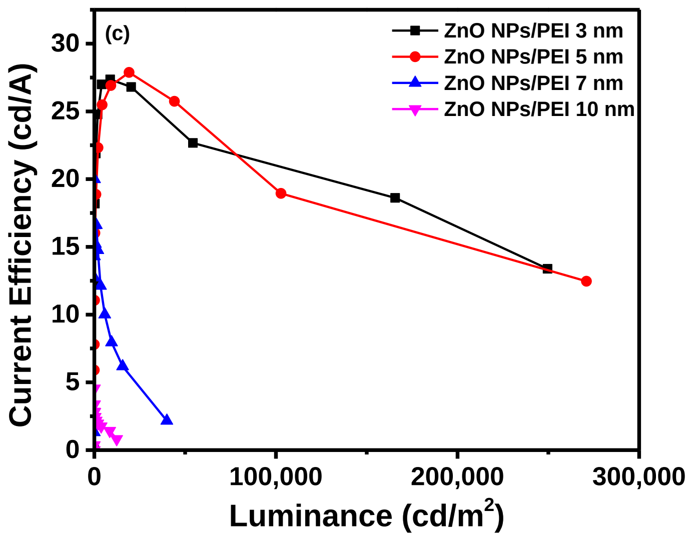

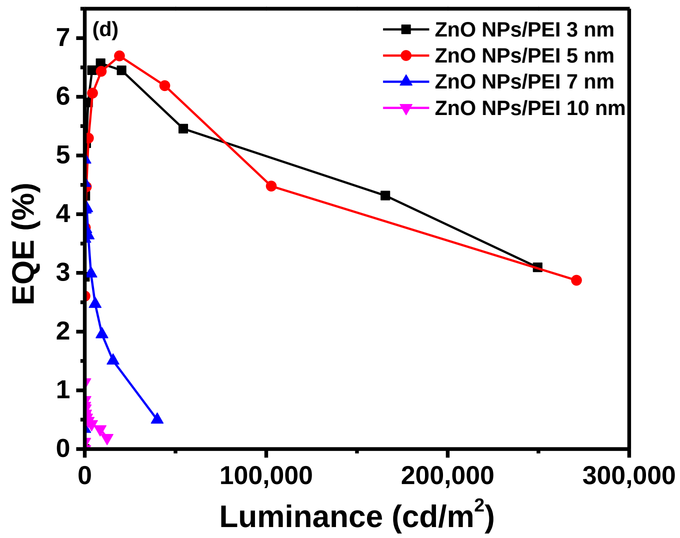

Figure S1. Electroluminescence characteristics of IQLEDs with ZnO NPs/PEI (3 nm), ZnO NPs/PEI (5 nm), ZnO NPs/PEI (7 nm), and ZnO NPs/PEI (10 nm): (a) current density-voltage curves, (b) luminance-voltage curves, (c) current efficiency-luminance curves, and (d) external quantum efficiency-luminance curves.

Table S1. The performance parameters of IQLEDs with ZnO NPs/PEI (3 nm), ZnO NPs/PEI (5 nm), ZnO NPs/PEI (7 nm), and ZnO NPs/PEI (10 nm) EILs.

Thickness of ZnO/PEI EIL (nm)	Current Density at 9V (mA/cm^2)	Maximum Luminance (cd/m^2)	Maximum Current Efficiency (cd/A)	Maximum EQE (%)
3	743.7	249,617	27.35	6.57
5	809.9	270,995	27.87	6.70
7	192.9	39,926	16.61	4.10
10	213.1	12,290	4.55	0.84

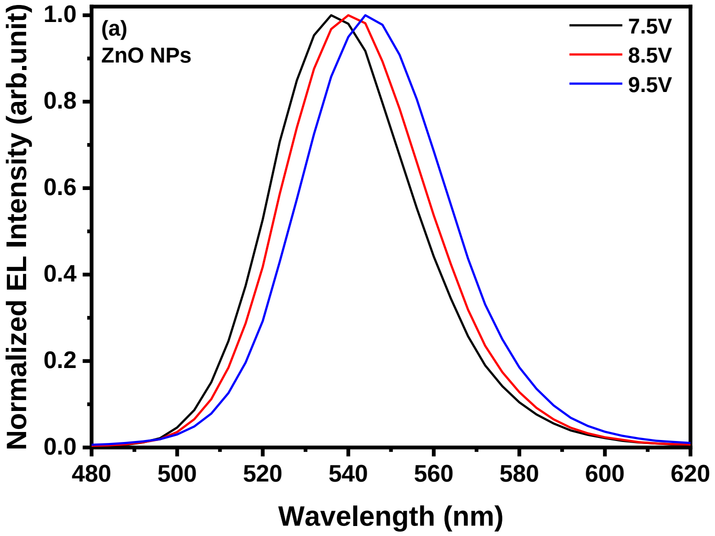

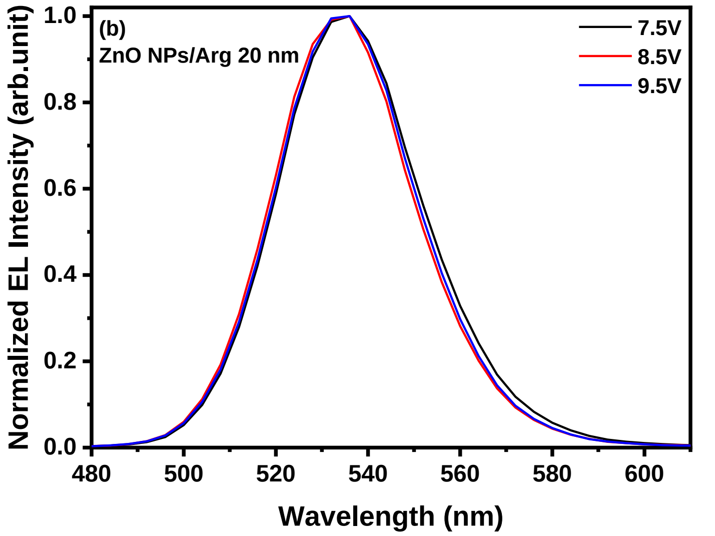

Figure S2. Electroluminescence spectra of IQLEDs, (a) with ZnO NPs EIL, (b) with ZnO NPs/Arg (20 nm) EIL at different applied voltages.

Table S2. The full widths at half maximum and peak positions of electroluminescence spectra of IQLEDs, (a) with ZnO NPs EIL, (b) with ZnO NPs/Arg (20 nm) EIL at different applied voltages.

EIL	Voltage (V)	7.5	8.5	9.5
ZnO NPs EIL	FWHM (nm)	39.50	39.90	40.76
	Peak (eV)	536	540	544
ZnO NPs/Arg (20 nm)	FWHM (nm)	36.40	35.91	36.00
	Peak (eV)	536	536	536
